# Adsorption behavior of Cr(VI) by biomass-based adsorbent functionalized with deep eutectic solvents (DESs)

**DOI:** 10.1186/s13065-022-00834-w

**Published:** 2022-06-02

**Authors:** Qian Li, Qing Huang, Xin Ya Pan, Hang Yu, Zi Tong Zhao

**Affiliations:** 1 Department of Chemistry & Life Science, Hubei University of Education, Wuhan, 430205 China; 2 Hubei Environmental Purification Material Science and Engineering Technology Research Center, Hubei University of Education, Wuhan, 430205 China; 3 Hubei Key Laboratory of Purification and Application of Plant Anti-Cancer Active Ingredients, Hubei University of Education, Wuhan, 430205 China; 4 Hubei Engineering Technology Center of Environmental Purification Materials, Hubei University of Education, Wuhan, 430205 China

**Keywords:** Cr(VI), Adsorption, Peanut shell, DESs

## Abstract

This study was aimed to evaluate the performance of DESs functionalized peanut shell (PSD) as biosorbent for removing Cr(VI) from water. The effects of pretreatment, initial concentration, adsorption temperature, kinetics, adsorption isotherm, and thermodynamics were investigated. Scanning electron microscopy (SEM) and Point of Zero charge (pHpzc) techniques were used for characterization of the adsorbents. The results showed that the rigid structure of peanut shell was broken down after DESs modification and the point of zero charge was 6.02 for peanut shell and 6.84 for PSD, which exhibited a slightly acid character. Based on the comparisons of linear and nonlinear analysis of four kinetic models and four isotherms, the pseudo-second-order kinetic model was found to be suitable for describing the adsorption process. The presence of a boundary effect was observed within the range of research, indicating that internal diffusion was not the only rate-controlling step. The equilibrium data were well represented by the Langmuir model rather than the Freundlich, Temkin, and Dubinin–Radushkevich models. The maximum capacity derived was 5.36 mg g^−1^. Changes in Gibb’s free energy, enthalpy, and entropy revealed that Cr(VI) adsorption onto modified peanut-shell powders was a spontaneous and endothermic process. However, the highest desorption efficiency was only 8.77% by using NaOH as a desorbing agent.

**Graphical abstract:**

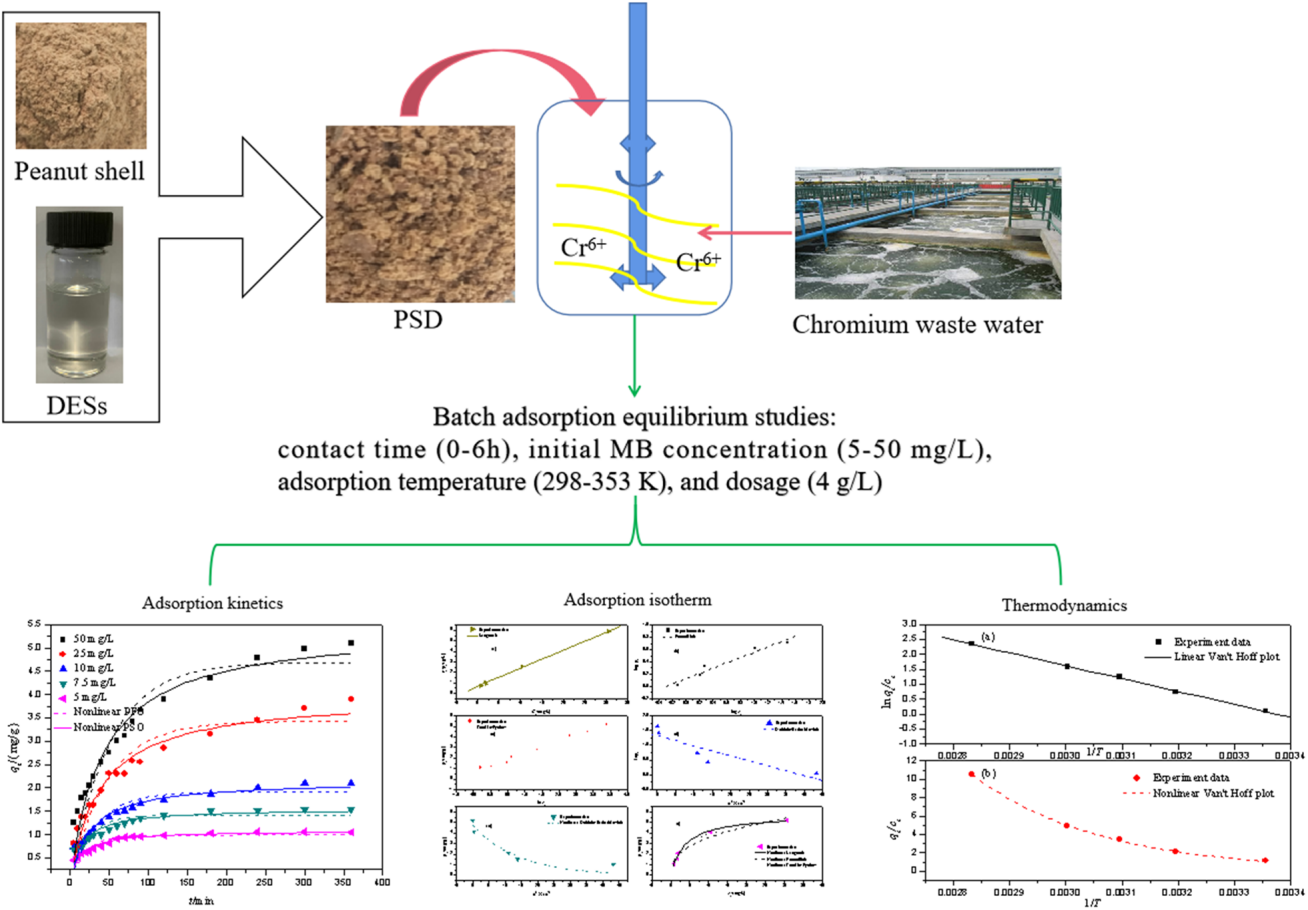

## Introduction

Heavy metal pollution caused by industrial operations is a global environmental issue. To minimize health risks, these contaminants should be removed from the aquatic ecosystem. Chromium, used in chrome plating, textile dyeing, wood preservation, and metal finishing, is classified as a priority pollutant by the Environmental Protection Agency (EPA) due to its high toxicity even at low concentrations [10, 12, 21]. Therefore, it is critical to remove chromium (VI) to make water safe.

There are several ways of eliminating heavy metals from wastewater. These include ion exchange, photocatalytic oxidation, electrochemical technologies, membrane separation processes and adsorption [2, 4, 16, 42]. El-Aryan et al. [13] prepared a type of ion exchange material based on zirconium phosphate and reported that the cation-exchanger showed a high selectivity for Pb(II) compared to other metal ions. Peng et al. [35] used lead sulfate precipitate chromium (VI) according to the solubility difference between lead sulfate and lead chromate and the precipitation efficiency was higher than 90%. Kumar et al. [23] studied the potentiality of the green emulsion liquid membrane (GELM) to extract hexavalent chromium and showed that extraction efficiency was up to 97% under the optimum process conditions. However, most of these removal technologies suffer from problems such as high capital investment and disposal of sludge, and they might not be feasible in small industries or at low concentrations of metal ions.

In contrast, adsorption is regarded one of the most practicable methods because of its low cost, ease of handling, high effectiveness, and environmental friendliness. A large number of adsorbents like activated carbon, have been developed to deal with wastewater [3]. According to recent research, bio-adsorbents made of cellulose have good adsorption characteristics in aqueous solutions due to their high surface area, high ion exchange capacity, and high affinity towards these pollutants. Several natural and synthetic bio-adsorbents have been found, including tea waste, mangosteen shell, oyster shell, walnut shell, Mosambi Fruit Peelings [9, 20, 22, 26, 32].

Taking into account the fact that China is one of the largest producers and consumers of peanuts in the world, the peanut shell is widely available. It has a porous texture and a large specific surface area, as well as functional groups such as hydroxyl, phenolic hydroxyl, carboxyl, and amino groups, making it easy to be modified. A study on this kind of materials as adsorbents is of great interest. However, many modification methods have drawbacks such as secondary pollution, high modification cost, and a tedious procedure. It is extremely important to develop a sustainable synthetic or modified technique for producing sustainable adsorbents with higher adsorption capacity.

Deep eutectic solvents (DESs) are a new class of compounds that can be used as solvents, reactants, or catalysts. In comparison to traditional ionic liquids (ILs), DESs have a number of advantages, including ease of preparation and availability from relatively low-cost components, low toxicity, biodegradability, and low vapor emissions [38]. The use of DESs as the pretreatment media appears promising in terms of its efficiency and biocompatibility. Sirviö et al. [37] employed choline chloride-urea to promote nanofibrillation of birch cellulose pulp, and the cellulose fibers disintegrated into nanofibril bundles with widths ranging from 15 to 200 nm and individual cellulose nanofibrils with widths ranging from 2–5 nm. Yu et al. used two types of DESs, choline chloride-oxalic acid dehydrate (CO) and choline chloride-urea (CU) for the nanofibrillation of ramie fibers (RFs). It turned out that CO DESs pretreatment improved nanofibrillation of RFs with low hemicellulose content substantially [43]. Kaur et al. [18] prepared choline chloride-based DESs as a functionalization agent for activated carbon derived from pumpkin seed shell for the uptake of doxycycline hydrochloride (DOX). Hussin et al. [17] studied the modification of low-cost biomass-based activated carbon with DESs to achieve high CO_2_ adsorption capacity, and a maximum adsorption capacity of 11.05 mg g^−1^ was attained at lower temperature.

The main advantages of choosing biomass-based adsorbents functionalized with DESs are sustainability and non-toxicity. Therefore, they have the potential to remove pollutants with no or less harmful effects on the environment compared to conventional treatment. In addition, converting biomass-based wastes into benign adsorbents is a practical solution to the disposal problem associated with these wastes. Nevertheless, to our knowledge, choline chloride-urea based DESs solutions used as a pretreatment for cellulose materials such as peanut shells have rarely been reported, and there is still a lack of detailed analysis regarding the adsorption of Cr(VI) from aqueous solution by using this type of DESs functionalized biosorbent. In this paper, the peanut shell was modified by using choline chloride-urea based DESs for the removal of Cr(VI) from aqueous solution. The kinetics, isotherms, thermodynamics, and desorption behavior related to the process were investigated. The results can be used as a reference for evaluating the ability of peanut shells to remove heavy metals within a green chemistry framework and promoting the use of sustainable alternative biomass-based materials.

## Materials and methods

### Preparation of adsorbents

The peanut-shell powder used in the experiments was purchased from local market (Wuhan, China). In order to eliminate the impurities, the peanut-shell was repeatedly washed, filtered, and dried. The dried samples were pulverized, sieved by a mesh size of 80, and were kept in desiccators until use.

### DESs pretreatment of peanut-shell powder

For pretreatment of peanut-shell powder, DESs were synthesized by heating choline chloride and urea with a molar ratio of 1:2 under constant stirring at 353 K for about 2 h until a clear and colorless liquid was obtained.

For optimizing the best pretreatment conditions, the peanut-shell powders were mixed with DESs at different ratios (0.5:20, 1:20, 3:20, 5:20) (W/W) and different pretreatment temperatures (298 K, 313 K, 323 K, 333 K, 353 K) for 2 h. The resulting mixture was then filtered, washed, dried and labeled as PSD. Analytical grade reagents were used in all cases.

### Characterization

The techniques used to characterize the PSD included Scanning Electron Microscope (FEI Quanta 200) and pH point of zero charge (pH_PZC_) [30].

### Adsorption of Cr(VI) on PSD

Adsorption experiments were carried out by making a series of 250 mL Cr(VI) solutions containing 4 g L^−1^ PSD. The initial pH was adjusted to 2 by adding 1 M HCl solutions. And then the suspension was stirred thoroughly at a certain adsorption temperature (298 K, 313 K, 323 K, 333 K, 353 K) for 6 h in a magnetic stirrer. After the reaction, the mixture was filtered, and the concentration of Cr(VI) in the filtrate was determined spectrophotometrically at 540 nm.

### Calculation of adsorption capacity

The percentage (%) of removal can be calculated as:

1 $$\eta =\frac{{C}_{0}-{C}_{e}}{{C}_{0}}\times 100\mathrm{\%}$$

The amount of Cr(VI) adsorbed *q*_t_ (mg g^−1^), at contacting time of *t* (min) can be calculated as:

2 $${q}_{t}=\frac{\left({C}_{0}-{C}_{t}\right)}{m}\times V$$

where *C*_o_ (mg L^−1^) is initial Cr(VI) concentration; *C*_t_ (mg L^−1^) is the residual Cr(VI) concentration at time *t*; *V* (L) is the volume of the solution, and *m* (g) is the dry weight of the adsorbent into each beaker.

### Adsorption kinetics

Kinetics studies were carried out by preparing a series of 250 mL Cr(VI) solutions containing 4 g L^−1^ PSD with the initial concentration range of 5–50 mg L^−1^ at different adsorption temperatures (298 K, 313 K, 323 K, 333 K, 353 K). The pH was adjusted to 2 using 1 M HCl. Samples were collected at different time intervals and analyzed. Different kinetic models were applied to fit the adsorption process.

#### The pseudo-first-order kinetic model

The non-linear and linear pseudo-first-order kinetic expressions can be given as follows:

3 $${q}_{t}={q}_{e}\left(1-{e}^{-{k}_{1}t}\right)$$

4 $$log\left({q}_{e}-{q}_{t}\right)=log{q}_{e}-\frac{{k}_{1}t}{2.303}$$

where *q*_e_ (mg g^−1^) and *q*_t_ (mg g^−1^) are amount of Cr(VI) adsorbed at equilibrium and at time *t* (min), respectively, and *k*_1_ (min^−1^) is the rate constant of pseudo-first order adsorption.

#### The pseudo-second-order kinetic model

The non-linear and linear pseudo-second-order kinetic expressions can be written as follows:

5 $${q}_{t}=\frac{{q}_{e}^{2}{k}_{2}t}{1+{q}_{e}{k}_{2}t}$$

6 $$\frac{t}{{q}_{t}}=\frac{1}{{k}_{2}{q}_{e}^{2}}+\frac{t}{{q}_{e}}$$

where *q*_e_ (mg g^−1^) and *q*_t_ (mg g^−1^) are the amount of Cr(VI) adsorbed at equilibrium and at time *t* (min), respectively, and *k*_2_ (g mg^−1^ min^−1^) is the rate constant of pseudo-second-order adsorption.

#### The Weber and Morris intraparticle diffusion model

The Weber and Morris intraparticle diffusion model can be expressed as follows:

7 $${q}_{t}={k}_{id}{t}^{1/2}+{C}_{1}$$

where *q*_t_ (mg g^−1^) is the amount of Cr(VI) adsorbed at time *t* (min), *k*_id_ (mg g^−1^ min^1/2^) is the rate constant of intraparticle diffusion, and *C*_1_ (mg g^−1^) is the intercept or the boundary layer thickness.

#### The Boyd kinetic model

The Boyd kinetic model can be described as follows:

8 $${B}_{t}=-0.4977-ln\left(1-\frac{{q}_{t}}{{q}_{e}}\right)$$

9 $${B}_{t}={k}_{b}t+{C}_{2}$$

where *B*_t_ is Boyd constant, *q*_e_ (mg g^−1^) and *q*_t_ (mg g^−1^) are the amount of Cr(VI) adsorbed at equilibrium and at time *t* (min), respectively, *k*_b_ (min^−1^) is the rate constant of Boyd kinetic model, and *C*_2_ is the intercept.

### Adsorption isotherm

The isotherm studies were carried out by varying the initial Cr(VI) concentrations from 5 to 50 mg L^−1^ at 298 K. The Langmuir, Freundlich, Temkin and Dubunin–Radushkevich adsorption models were applied to characterize the adsorption mechanism.

#### The Langmuir isotherm

The non-linear and linear forms of Langmuir isotherm can be expressed as:

10 $${q}_{e}=\frac{{{q}_{m}K}_{L}{C}_{e}}{1+{K}_{L}{C}_{e}}$$

11 $$\frac{{C}_{e}}{{q}_{e}}=\frac{{K}_{L}}{{q}_{m}}+\frac{{C}_{e}}{{q}_{m}}$$

where *q*_e_ (mg g^−1^) is the amount of Cr(VI) adsorbed at equilibrium, *C*_e_ (mg L^−1^) is the equilibrium concentration of Cr(VI), *q*_m_ (mg g^−1^) is maximum adsorption capacity, *K*_L_ (mg L^−1^) is the Langmuir constant.

12 $$\mathrm{The\, separation\, factor }\,{R}_{L}=\frac{1}{1+{C}_{0}/{K}_{L}}$$

where *C*_0_ (mg L^−1^) is the lowest initial concentration of Cr(VI), and *K*_L_ (mg L^−1^) is the Langmuir constant.

#### The Freundlich isotherm

The non-linear and linear forms of Freundlich isotherm can be written as:

13 $${q}_{e}={{K}_{F}{C}_{e}}^{1/n}$$

14 $$log{q}_{e}=\frac{log{C}_{e}}{n}+log{K}_{F}$$

where *q*_e_ (mg g^−1^) is the amount of Cr(VI) adsorbed at equilibrium, *C*_e_ (mg L^−1^) is the equilibrium concentration of Cr (VI), *n* is related to the intensity of adsorption, *K*_F_ (mg g^−1^) is the Freundlich constant.

#### The Temkin–Pyzhev isotherm

The non-linear and linear forms of Temkin–Pyzhev isotherm can be shown as:

15 $${q}_{e}=\frac{RTlnA{C}_{e}}{{b}_{T}}$$

16 $${q}_{e}=\frac{RTln{C}_{e}}{{b}_{T}}+\frac{RTlnA}{{b}_{T}}$$

where *q*_e_ (mg g^−1^) is the amount of Cr(VI) adsorbed at equilibrium, *C*_e_ (mg L^−1^) is the equilibrium concentration of Cr(VI), *b*_T_ (J mol^−1^) is the Temkin–Pyzhev isotherm constant, *A* (L mg^−1^) is Temkin–Pyzhev isotherm equilibrium binding constant, *R* (8.314 J mol^−1^ K^−1^) is the universal gas constant, *T* (K) is the absolute temperature.

#### The Dubinin–Radushkevich isotherm

The non-linear and linear forms of Dubinin–Radushkevich isotherm can be described as:

17 $${q}_{e}={q}_{m}{e}^{-\beta {\varepsilon }^{2}}$$

18 $$ln{q}_{e}=-\beta {\varepsilon }^{2}+ln{q}_{m}$$

19 $$\varepsilon =RTln\left(1+\frac{1}{{C}_{e}}\right)$$

20 $$E=\frac{1}{{\left(2\beta \right)}^{1/2}}$$

where *q*_e_ (mg g^−1^) is the amount of Cr (VI) adsorbed at equilibrium, *q*_m_ (mg g^−1^) is maximum adsorption capacity, *C*_e_ (mg L^−1^) is the equilibrium concentration of Cr (VI), *ε* (J mol^−1^) is the Potential of Polanyi, *β* (mol^2^ kJ^−2^) is a constant related to the mean free energy of adsorption per mole of an adsorbate, *R* (8.314 J mol^−1^ K^−1^) is the universal gas constant, *T* (K) is the absolute temperature, and *E* (kJ mol^−1^) is the mean adsorption energy.

### Desorption experiments

The desorption experiments were carried out after adsorption equilibrium by using four different desorbing agents: acetic acid solution, sodium hydroxide solution, EDTA solution, and deionized water. Briefly, 0.2 g of Cr(VI) loaded PSD was mixed with 50 mL of desorbing agent within a pH range of 2–12 at 298 K. Then the desorbed Cr(VI) concentration was analyzed spectrophotometrically after the suspension was stirred for a fixed time. The desorption efficiency of Cr(VI) can be calculated as follows:

21 $${\eta }^{^{\prime}}=\frac{{C}_{\mathrm{t}}^{\mathrm{^{\prime}}}{V}^{\mathrm{^{\prime}}}}{{{q}_{e}m}^{\mathrm{^{\prime}}}}\times 100\mathrm{\%}$$

where *C*_t_^ʹ^ (mg L^−1^) is the desorbed Cr(VI) concentration at time *t* (min^−1^); *V*ʹ (L) is the volume of the desorption solution, and *m*ʹ (g) is the dry weight of the Cr(VI) loaded PSD into each beaker. *q*_e_ (mg g^−1^) is the equilibrium adsorbed amount.

## Results and discussion

### The effect of pretreatment on adsorption

#### Effect of the ratio of peanut shell powder to DESs

**Fig. 1:**
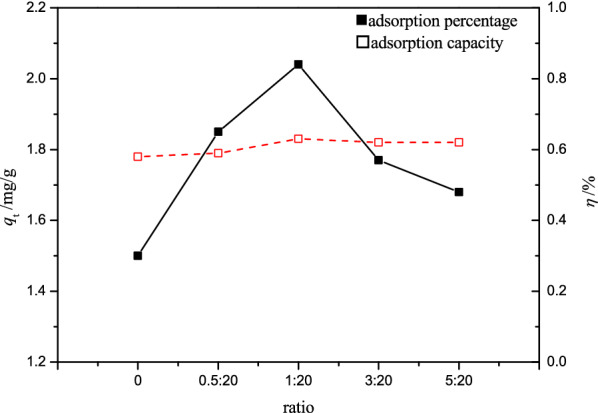
Effect of pretreatment ratio on Cr(VI) adsorption by PSD

At pretreatment temperature of 353 K, the effect of ratio on Cr(VI) removal under the adsorption conditions of pH 2, adsorbent dose of 4 g L^−1^, contact time of 2 h, 298 K, is shown in Fig. 1. As the ratio of peanut shell powder to DESs increased, the adsorption percentage did not change much, but the adsorption capacity first increased and then decreased. That might be due to the fact that as the amount of peanut shell powder increased, the powders were distributed unevenly on the surface of the DESs, resulting in insufficient contact and an unsatisfactory modification effect. Furthermore, excessive peanut shell powder made mixing more difficult, leading to poor interaction between the peanut shell and DESs. As a result, the ratio of 1:20 was preferred.

#### Effect of pretreatment temperature

**Fig. 2:**
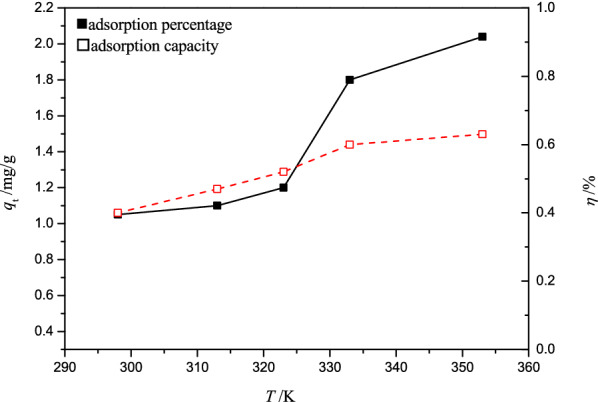
Effect of pretreatment temperature on Cr(VI) adsorption by PSD

At the ratio of peanut shell powder to DESs of 1:20, the effect of pretreatment temperature on Cr(VI) removal under the adsorption conditions of pH 2, adsorbent dose of 4 g L^−1^, contact time of 2 h, 298 K, is shown in Fig. 2.

As seen from Fig. 2, the adsorption percentage and adsorption capacity of peanut shell powder for Cr(VI) increased with the increase of the pretreatment temperature. This result can be attributed to the viscosity of DESs, which was lower at higher temperatures and thus facilitated the contact with peanut shell powder. In addition, higher temperatures also provided more energy for the dissolution of peanut shell powder in DESs, which enhanced the breakage of hydrogen bonds in its internal cellulose structure. To reduce energy consumption, the pretreatment temperature of 353 K was acceptable.

### Kinetics of adsorption

Adsorption kinetics describes the change of adsorption amount with time, which can be used to analyze the adsorption rate and provide a reference for the design of sewage treatment equipment in terms of minimizing contact time and cost [29].

**Fig. 3:**
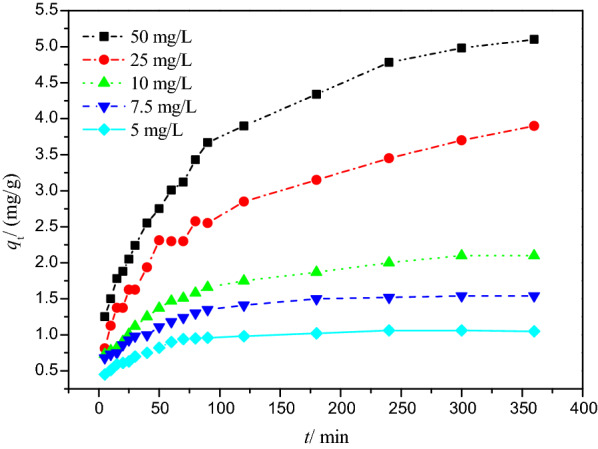
Cr(VI) adsorption at different initial concentrations

**Fig. 4:**
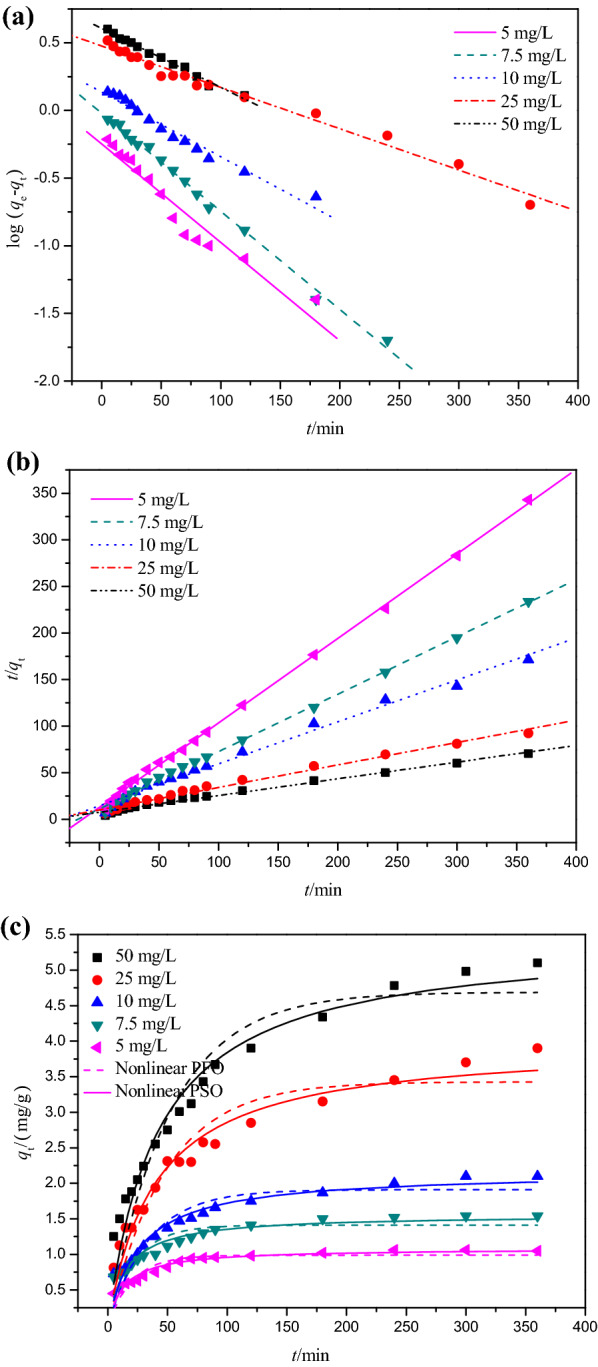
**a** Linear pseudo-first-order kinetic model for adsorption of Cr(VI) ion at different initial concentrations; **b** Linear pseudo-second-order kinetic model for adsorption of Cr(VI) ion at different initial concentrations; **c** Nonlinear pseudo-first-order and pseudo-second-order kinetic models for adsorption of Cr(VI) ion at different initial concentrations

**Fig. 5:**
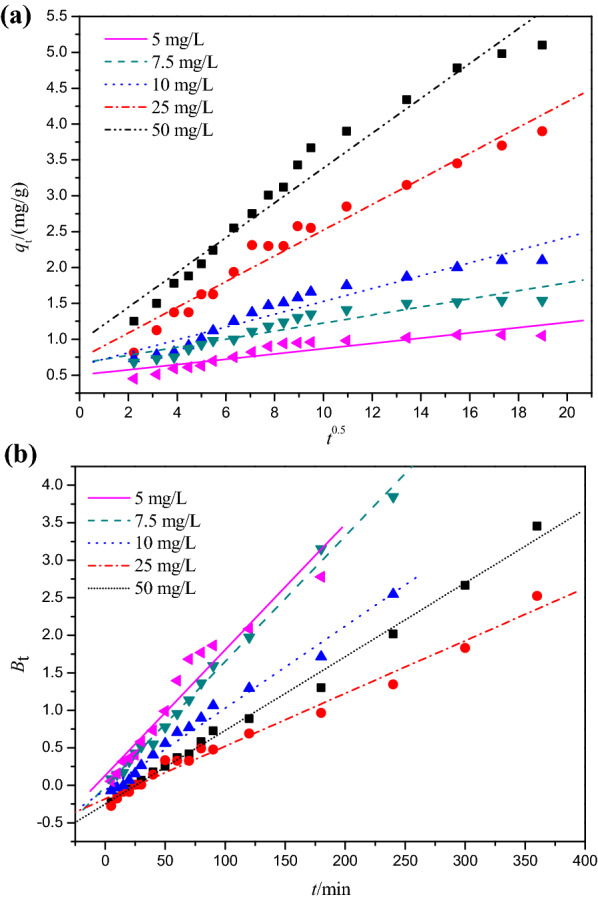
**a** Webber–Morris intraparticle diffusion model for adsorption of Cr(VI) ion at different initial concentrations; **b** Boyd film diffusion model for adsorption of Cr(VI) ion at different initial concentrations

The kinetics of Cr(VI) adsorption on DESs modified peanut-shell powders (PSD) at different initial concentrations is shown in Fig. 3 and evaluated by using the linear and nonlinear pseudo-first-order, pseudo-second-order, intraparticle diffusion, and Boyd models (Figs. 4 and 5). The fitting results derived from these models are listed in Tables 1 and 2.

**Table 1:** (a) Parameters of the linear and nonlinear pseudo-first model for Cr(VI) adsorption on PSD at different initial concentrations; (b) Parameters of the linear and nonlinear pseudo-second order model for Cr(VI) adsorption at different initial concentrations

(a)							
*C*_0_/mg L^−1^	*q*_e,exp_/mg g^−1^	Pseudo first order					
		Linear form			Nonlinear form		
		*q*_e,cal_/mg g^−1^	*k*_1_/min^−1^	*R*^2^	*q*_e,cal_/mg g^−1^	*k*_1_/min^−1^	*R*^2^
5	1.06	0.56	1.68 × 10^–2^	0.9686	0.99	4.95 × 10^–2^	0.8232
7.5	1.54	0.95	1.68 × 10^–2^	0.9977	1.41	4.47 × 10^–2^	0.7521
10	2.1	1.37	1.11 × 10^–2^	0.9842	1.91	2.92 × 10^–2^	0.853
25	4.1	2.99	7.08 × 10^–3^	0.9917	3.43	2.08 × 10^–2^	0.8879
50	5.2	4.03	1.01 × 10^–2^	0.9948	4.69	1.96 × 10^–2^	0.8996

(b)							
*C*_0_/mg L^−1^	*q*_e,exp_/mg g^−1^	Pseudo second order					
		Linear form			Nonlinear form		
		*q*_e,cal_/mg g^−1^	*k*_2_/g^−1^ mg^−1^ min^−1^	*R*^2^	*q*_e,cal_/mg g^−1^	*k*_2_/g^−1^ mg^−1^ min^−1^	*R*^2^
5	1.06	1.1	6.38 × 10^–2^	0.9995	1.09	6.92 × 10^–2^	0.9336
7.5	1.54	1.62	3.40 × 10^–2^	0.9992	1.56	4.26 × 10^–2^	0.8979
10	2.1	2.22	1.39 × 10^–2^	0.9951	2.16	1.81 × 10^–2^	0.9362
25	4.1	4.16	5.66 × 10^–3^	0.9941	3.98	6.39 × 10^–3^	0.9525
50	5.2	5.58	4.24 × 10^–3^	0.9954	5.45	4.39 × 10^–3^	0.9541

**Table 2:** Parameters of Webber–Morris intraparticle and Boyd film diffusion models for Cr(VI) adsorption on PSD at different initial concentrations

*C*_0_/ mg L^−1^	*k*_id_/ mg g^−1^ min^1/2^	*C*_1_	*R*^2^	*k*_*b*_/ min^−1^	*C*_2_	*R*^2^
5	3.68 × 10^–2^	0.5	0.8958	1.68 × 10^–2^	0.13	0.9686
7.5	5.63 × 10^–2^	0.66	0.937	1.67 × 10^–2^	− 0.02	0.9977
10	8.94 × 10^–2^	0.63	0.9618	1.09 × 10^–2^	− 6.46	0.9919
25	0.18	0.73	0.9819	7.03 × 10^–3^	− 0.18	0.9917
50	0.24	0.96	0.9828	9.82 × 10^–3^	− 0.25	0.9972

Correlation coefficients (*R*^2^) were used to indicate the consistency between experimental results and model predicted values. At various Cr(VI) concentrations, the correlation coefficient values (*R*^2^) for the linear and nonlinear pseudo-second-order models were higher than those obtained from the linear and nonlinear pseudo-first-order models, indicating that the pseudo-second-order model can effectively describe the Cr(VI) adsorption kinetics.

Furthermore, compared with the results from the linear and nonlinear pseudo-first-order models, the *q*_cal_ values predicted from the linear pseudo-second-order model (1.1, 1.62, 2.22, 4.16, and 5.58 mg g^−1^) and from the nonlinear pseudo-second-order model (1.09, 1.56, 2.16, 3.98, and 5.45 mg g^−1^) for different Cr(VI) concentrations were much closer to the experimental values *q*_exp_ (1.06, 1.54, 2.1, 4.1, 5.2 mg g^−1^).

These results confirmed that the pseudo-second-order kinetic model was applicable and implied that electron sharing or electron transfer between PSD and Cr(VI) might be involved in this process. Similar kinetic fitting results have been reported for Cr(VI) adsorption [6, 19].

The intraparticle diffusion and Boyd models were used in this study to further understand the potential diffusion mechanism. Table 2 shows the fitting results of intraparticle diffusion and Boyd models for Cr(VI) adsorption onto PSD. Generally, if intraparticle diffusion is involved in the adsorption process, adsorption capacity should be plotted against the square root of time linearly, and if the plot passes through the origin, intraparticle diffusion is the only rate determining step. If the plot doesn’t pass through the origin, there might be some degree of boundary layer control, and the values of the intercept *C* can be used to estimate the thickness of the boundary layer, i.e., the larger the intercept, the greater the boundary layer effect [7, 33].

In this study, the intraparticle diffusion model demonstrated that internal diffusion was not the only rate controlling step because none of the plots passed the origin (Fig. 5a). This illustrated that there were other mechanisms involved in addition to intraparticle diffusion.

Five fitting curves of Boyd model, as depicted in Fig. 5b, followed a linear pattern, but none of them passed through the origin. As a result, boundary effect was important in the adsorption of Cr(VI) by PSD, which was consistent with the findings of the intraparticle diffusion model. The *R*^2^ values of the Boyd model at 5, 7.5, 10, 25, and 50 mg L^−1^ were 0.9686, 0.9977, 0.9919, 0.9917, and 0.9972, respectively, showing the applicability of this model.

As a result of these findings, adsorption behaviors of Cr(VI) onto PSD were complex, involving multiple rate-controlling steps.

### Adsorption isotherms

**Fig. 6:**
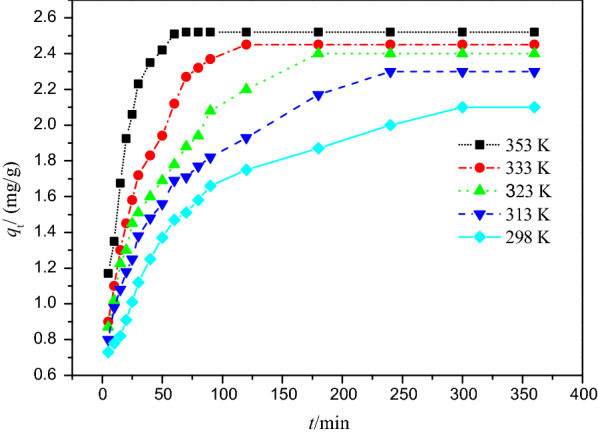
Cr(VI) adsorption at different temperatures

**Fig. 7:**
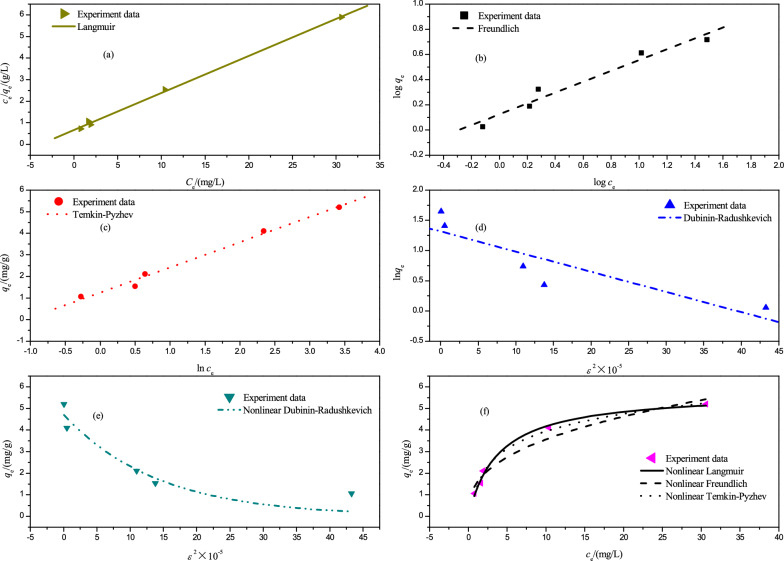
**a** Linear Langmuir isotherm for Cr(VI) adsorption on PSD at 298 K; **b** Linear Freundlich isotherm for Cr(VI) adsorption on PSD at 298 K; **c** Linear Temkin-Pyzhev isotherm for Cr(VI) adsorption on PSD at 298 K; **d** Linear Dubinin–Radushkevich isotherm for Cr(VI) adsorption on PSD at 298 K; **e** Nonlinear Dubinin–Radushkevich isotherm for Cr(VI) adsorption on PSD at 298 K; **f** Nonlinear Langmuir, Freundlich, and Temkin–Pyzhev isotherms for Cr(VI) adsorption on PSD at 298 K

In order to investigate the equilibrium characteristics of Cr(VI) adsorption onto PSD samples, more adsorption experiments were carried out under different temperatures (Fig. 6). As seen in Fig. 6, the equilibrium adsorption capacity was higher at higher temperatures. The linear and nonlinear Langmuir, Freundlich, Dubinin Radushkevich and Temkin isotherm models were applied to analyze these adsorption behaviors (Fig. 7).

As shown in Table 3, the adsorption process was appropriately represented by the linear or nonlinear Langmuir models with *R*^2^ values of 0.99, which were greater than the other three isotherm models. In addition, the maximum Cr(VI) adsorption capacity obtained from linear and nonlinear Langmuir models were 5.84 and 5.77 mg g^−1^, respectively. Moreover, according to the value of *R*_L_, the adsorption of Cr(VI) onto PSD was favorable [15]. This result was in accordance with other reports obtained by Liang and Tsamo [27, 39].

**Table 3:** Parameters of different linear and nonlinear isotherms for Cr(VI) adsorption on PSD at 298 K

Isotherm	Parameters			
Langmuir	Linear form			
	*q*_m_/mg g^−1^	*K*_*L*_/mg L^−1^	*R*_L_	*R*^2^
	5.84	3.92	0.07	0.999
	Nonlinear form			
	*q*_m_/mg g^−1^	*K*_*L*_/mg L^−1^	*R*_L_	*R*^2^
	5.77	3.84	0.07	0.9918
Freundlich	Linear form			
	*K*_F_/mg g^−1^	*n*		*R*^2^
	1.33	2.32		0.9786
	Nonlinear form			
	*K*_F_/mg g^−1^	*n*		*R*^2^
	1.5	2.66		0.9618
Temkin–Pyzhev	Linear form			
	*b*_T_/kJ mol^−1^	*A*/L mg^−1^		*R*^2^
	2.12	2.93		0.9948
	Nonlinear form			
	*b*_T_/kJ mol^−1^	*A*/L mg^−1^		*R*^2^
	2.12	2.93		0.9897
Dubinin–Radushkevich	Linear form			
	*E*/kJ mol^−1^	*q*_m_/mg g^−1^		*R*^2^
	1.22	3.73		0.885
	Nonlinear form			
	*E*/kJ mol^−1^	*q*_m_/mg g^−1^		*R*^2^
	2.65	4.72		0.9028

The Freundlich model didn’t match the experimental data as well as the Langmuir model. The heterogeneity factor (*n*) of the linear and nonlinear Freundlich isotherms were derived to be 2.32 and 2.66, confirming that the adsorption was favorable.

In this investigation, the Temkin–Pyzhev model appeared to have a decent fitting result, indicating that electrostatic might be involved in this process. The value of *E* derived from the D–R model was less than 8 kJ mol^−1^, indicating the physical nature of this process. However, the *R*^2^ value for the D–R model was lower than for other models, showing that the D–R model was not appropriate for describing Cr(VI) adsorption onto PSD.

### Adsorption thermodynamics

The thermodynamic parameters ${\Delta G}^{\ominus }$, ${\Delta H}^{\ominus }$, and ${\Delta S}^{\ominus }$ of the chromium adsorption by PSD can be calculated by the following equations:

22 $${\Delta G}^{\ominus }={\Delta H}^{\ominus }-T{\Delta S}^{\ominus }$$

23 $${\Delta G}^{\ominus }=-RTln{K}_{\mathrm{e}}$$

24 $${K}_{\mathrm{e}}=\frac{{q}_{e}}{{C}_{\mathrm{e}}}=exp\left(\frac{{\Delta S}^{\ominus }}{R}-\frac{{\Delta H}^{\ominus }}{RT}\right)$$

25 $$ln\frac{{q}_{e}}{{C}_{\mathrm{e}}}=-\frac{{\Delta H}^{\ominus }}{RT}+\frac{{\Delta S}^{\ominus }}{R}$$

where *K*_e_ is the distribution coefficient of the adsorbate, and *q*_e_ (mg g^−1^) and *C*_e_ (mg L^−1^) are the equilibrium concentration of Cr(VI) on the adsorbent and in the solution, respectively. *R* (8.314 J mol^−1^ K^−1^) is the universal gas constant and *T* (K) is the temperature.

**Fig. 8:**
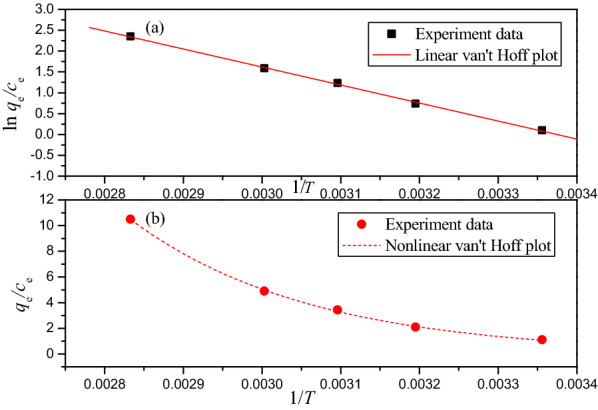
**a** Linear van’t Hoff plot of Cr(VI) adsorption on PSD; **b** Nonlinear van’t Hoff plot of Cr(VI) adsorption on PSD

The linear and nonlinear van’t Hoff plot are shown in Fig. 8 and the fitting results are summarized in Table 4. The ${\Delta G}^{\ominus }$ values were derived to be negatively ranging between − 0.16 and − 6.9 kJ mol^−1^ for various temperatures, confirming that this adsorption process was spontaneous and feasible in the range of 298–353 K.

**Table 4:** Thermodynamic parameters for the Cr(VI) adsorption on PSD

Linear results							
${\Delta H}^{\ominus }$/kJ mol^−1^	${\Delta S}^{\ominus }$/J mol^−1^ K^−1^	${\Delta G}^{\ominus }$/kJ mol^−1^					R^2^
		298 K	313 K	323 K	333 K	353 K	
35.94	121.29	− 0.2	− 2.02	− 3.23	− 4.45	− 6.87	0.9995
Nonlinear results							
${\Delta H}^{\ominus }$/kJ mol^−1^	${\Delta S}^{\ominus }$/J mol^−1^ K^−1^	${\Delta G}^{\ominus }$/kJ mol^−1^					R^2^
		298 K	313 K	323 K	333 K	353 K	0.9996
36.36	122.55	− 0.16	− 2	− 3.22	− 4.45	− 6.9	

In addition, the change in Gibbs free energy decreased with increasing temperature, indicating that the adsorption was endothermic. The positive value of the enthalpy change ${\Delta H}^{\ominus }$ (35.94 and 36.36 kJ mol^−1^) supported the endothermic character of the adsorption process. Other researchers have also reported similar results [1, 11].

The entropy change, ${\Delta S}^{\ominus }$ were determined to be 121.29 and 122.55 J mol^−1^ K^−1^ from linear and nonlinear analysis, respectively. The positive value of ${\Delta S}^{\ominus }$ revealed this process caused the increase in disorder of the system.

### Desorption experiments

Table 5 shows the effect of pH on Cr(VI) desorption efficiency. As the pH increased from 2 to 12, the desorption efficiency increased from 0.37% to 2.89%. The best desorption was achieved at pH of 12. The comparison of the efficiency of different desorbing agents is also shown in Table 5, and the highest desorption efficiency was about 8.77% by using hydroxide solution in the present study. However, the desorption efficiency of PSD was still very low under all the experiment conditions, indicating that the adsorption between PSD and chromium ions was not a simple physical adsorption, but the result of chemical adsorption and other mechanisms. In addition, some Cr(VI) might be reduced to Cr III), which cannot be diluted with base, resulting in poor desorption efficiency [28]. Similar studies in China have shown that it was difficult for Cr(VI) to be completely desorbed from some cellulosic-based biosorbents like tea stalks [40]. Considering the reduction reaction and the multiple forms of Cr(VI) in solution, the desorption mechanism of this type of biosorbents needed to be further studied.

**Table 5:** Results of desorption of PSD

No	Desorbing agents	Desorption efficiency/%
1	Deionized water (pH = 2)	0.37
2	Deionized water (pH = 4)	0.23
3	Deionized water (pH = 6)	0.59
4	Deionized water (pH = 10)	2.22
5	Deionized water (pH = 12)	2.89
6	0.1 mol L^−1^ HAc	0.26
7	0.1 mol L^−1^ NaOH	8.77
8	0.1 mol L^−1^ EDTA	0.46

### Possible adsorption mechanism

**Fig. 9:**
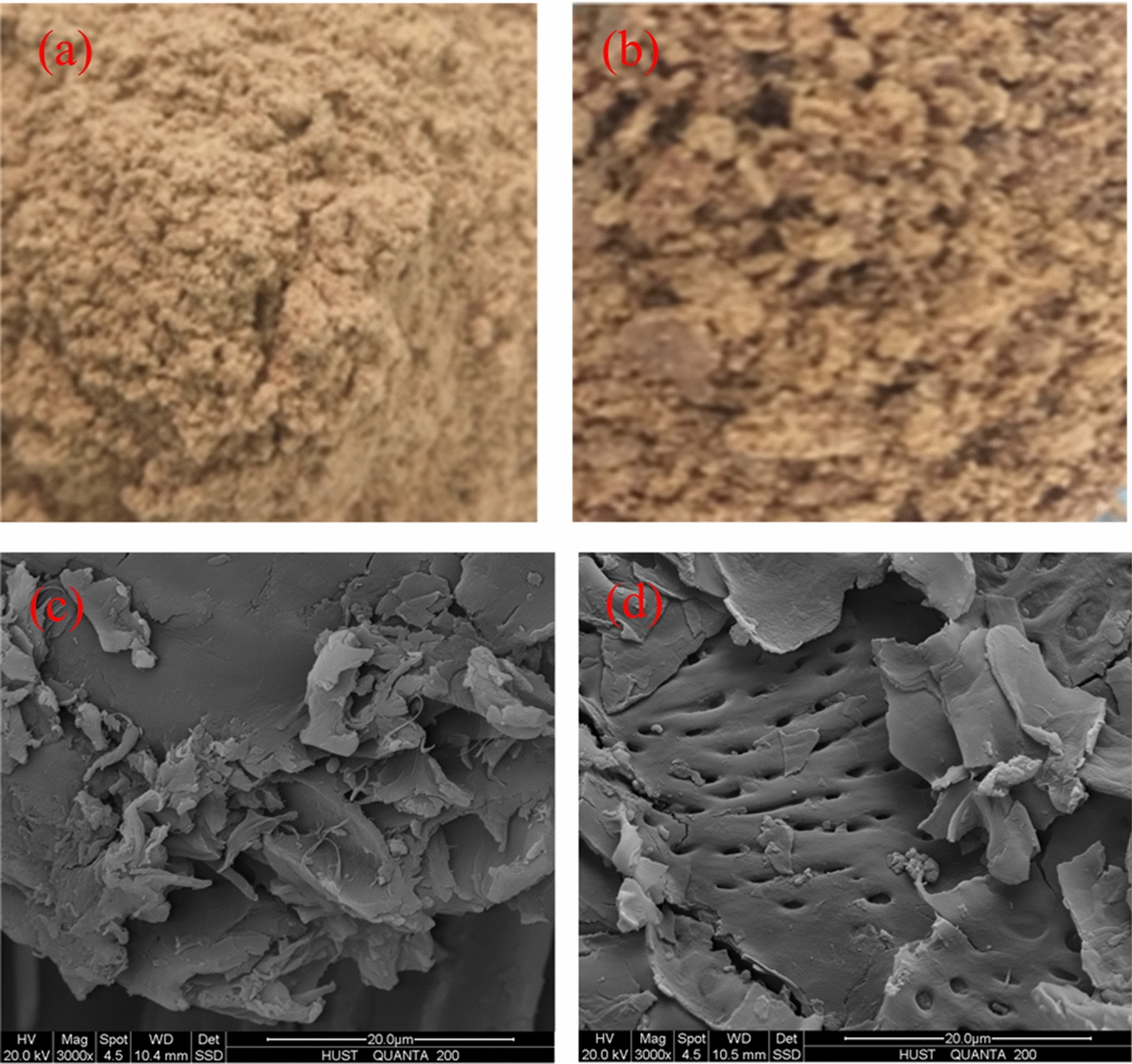
Optical images: **a** original peanut shell and **b** DESs pretreated peanut shell; SEM images at different magnifications: **c** original peanut shell (3000×) and **d** DESs pretreated peanut shell (3000×)

To investigate the possible mechanism of Cr(VI) adsorption on PSD, morphology and surface charge of the adsorbents were evaluated as discussed below. The optical and SEM images of peanut shell and PSD are shown in Fig. 9.

As can be seen in Fig. 9a and b, the dispersed granular form of peanut shell was maintained with no macro changes. However, based on SEM images shown in Fig. 9c and d, a large portion of the cellulosic structure of the peanut shell was unexposed and covered with lignin and hemicelluloses before DESs treatment, whereas the rigid connecting structure between lignin and hemicelluloses was broken down after DESs treatment, showing a more heterogeneous and porous structure. Additionally, Li et al. [25] and Zhang et al. [44] reported that the DESs can form hydrogen bonds with the hydroxyl groups in the molecular chains of cellulose, thereby weakening the intramolecular or intermolecular hydrogen bonds of cellulose and releasing more hydroxyl groups. Therefore, more adsorption sites were available on PDS because of its rough surface and increasing functional groups.

**Fig. 10:**
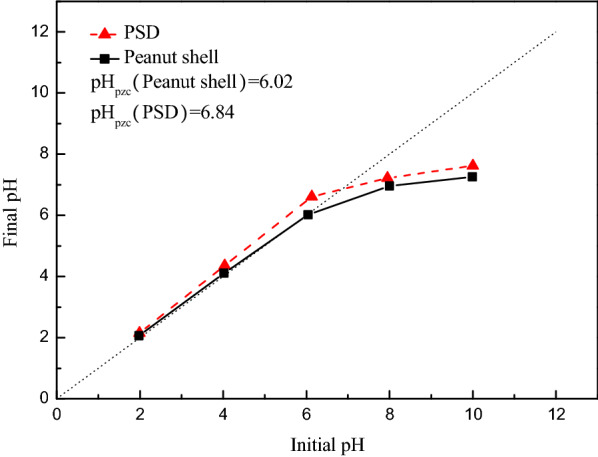
The plot of pH_pzc_

The surface charge of PSD was evaluated by measuring the pH at the point of zero charge (pH_PZC_). Generally, the adsorbent will exhibit better affinities for anions at pH < pH_PZC_ and vice versa. Figure 10 shows the plot of pH_final_ versus pH_initial_ for PSD. It can be seen that the pH_PZC_ value obtained for peanut shell was approximately 6.02, which was consistent with the value obtained by Banerjee et al. [5] and Gama et al. [14]. However, the pH_PZC_ value of PSD was up to 6.84. The increase in the pH_PZC_ of peanut shell might be explained by the fact that DESs treatment lead to an increase in the number of N-functional groups [34, 41]. It was expected that the adsorption of Cr(VI) could be enhanced at the experimental pH (pH = 2) due to electrostatic interaction between the main species of Cr(VI), Cr_2_O_7_^2−^, HCr_2_O_7_^−^ and nitrogen-containing functional groups such as –NH_3_^+^ on the surface of PSD [24, 36]. Similar results were reported by Ma et al., in which their research observed that the electrostatic attraction between Cr(VI) and protonated amino groups increased the Cr(VI) removal efficiencies under acidic conditions [31]. In addition, the composition of peanut shell comprises abundant oxygen-containing functional groups such as –COOH and –OH. Because of the protonation effect, the surface of adsorbents will have positive charges below pH_PZC_, thus improving the attraction for anions [8].

Therefore, although this process followed the pseudo-second-order model, it was not a normal chemical process since effective physical interaction can also lead to this observation. It appeared that Cr(VI) adsorption by PSD was physicochemical adsorption rather than a pure physical or chemical process.

## Conclusion

The application of DESs modified peanut-shell powders for removing Cr(VI) from aqueous solution was discussed. The removal of Cr(VI) was found to be dependent on the initial concentration and temperature. The removal percentage was decreased with increase in initial concentration but increased with increase in temperature. Kinetic studies showed that the pseudo-second-order model was best fitted for the sorption of Cr(VI) onto PSD at various initial concentrations. The Langmuir model was the best at describing the equilibrium data as compared to the Freundlich, Temkin–Pyzhev, and Dubinin–Radushkevich models over the studied temperature range. The adsorption was favorable as indicated by the separation factor (*R*_L_). The adsorption mechanism between Cr(VI) onto PSD was discovered to be a spontaneous and endothermic process by thermodynamic analysis. Several mechanisms like hydrogen bonding and electrostatic interaction were involved in the adsorption process. However, the desorption efficiency was relatively low, which needed to be further studied.

## Data Availability

The datasets used or analyzed during the current study are available from the corresponding author on reasonable request.

## References

[1] Ali IH, Alrafai HA (2016). Kinetic, isotherm and thermodynamic studies on biosorption of chromium(VI) by using activated carbon from leaves of Ficusnitida. Chem Cent J.

[2] Anarakdim K, Gutierrez G, Cambiella A, Senhadji-Kebiche O, Matos M (2020). The effect of emulsifiers on the emulsion stability and extraction efficiency of Cr(VI) using emulsion liquid membranes (ELMs) formulated with a green solvent. Membranes.

[3] Anfar Z, AitAhsaine H, Zbair M, Amedlous A, Ait El Fakir A, Jada A, El Alem N (2019). Recent trends on numerical investigations of response surface methodology for pollutants adsorption onto activated carbon materials: a review. Crit Rev Environ Sci Technol.

[4] Azimi A, Azari A, Rezakazemi M, Ansarpour M (2017). Removal of heavy metals from industrial wastewaters: a review. ChemBio-Eng Rev.

[5] Banerjee M, Basu RK, Das SK (2018). Cu(II) removal using green adsorbents: kinetic modeling and plant scale-up design. Environ Sci Pollut Res.

[6] Bhattacharya AK, Naiya TK, Mandal SN, Das SK (2007). Adsorption, kinetics and equilibrium studies on removal of Cr(VI) from aqueous solutions using different low-cost adsorbents. Chem Eng J.

[7] Bhattacharyya KG, Sharma A (2004). Azadirachtaindica leaf powder as an effective biosorbent for dyes: a case study with aqueous Congo Red solutions. J Environ Manage.

[8] Carvalho JT, Milani PA, Consonni JL, Labuto G, Carrilho EN (2020). Nanomodified sugarcane bagasse biosorbent: synthesis, characterization, and application for Cu(II) removal from aqueous medium. Environ Sci Pollut Res.

[9] Charnkeitkong P, Sripiboon S (2021). Chemical activation of Garcinia mangostana mangosteen shell with acid-base for hexavalent chromium adsorption. Key Eng Mater.

[10] de Andrade Neto JC, Pereira GJ, Morandim-Giannetti AD (2020). Chitosan and corn stover derivative bioadsorbent: characterization and application in hexavalent chromium adsorption processes. Cellulose.

[11] Dim PE, Mustapha L, Termtanun M, Okafor J (2021). Adsorption of chromium (VI) and iron (III) ions onto acid-modified kaolinite: Isotherm, kinetics and thermodynamics studies. Arab J Chem.

[12] Dong L, Deng R, Xiao H, Chen F, Zhou Y, Li J, Chen S, Yan B (2019). Hierarchical polydopamine coated cellulose nanocrystal microstructures as efficient nanoadsorbents for removal of Cr(VI) ions. Cellulose.

[13] El-Aryan YF, Alqahtany FZ (2022). Removal of some heavy metals from wastewater using hydrothermally synthesized zirconium phosphate as ion exchange material. Russ J Phys Chem A.

[14] Gama BM, Barbosa CB, Rodríguez-Díaz JM, Sales DC, Duarte MM (2021). Adsorption and recovery of cadmium and copper ions in mono and bicomponent systems using peanut shells biochar as a sustainable source: model development. Chem Eng Commun.

[15] Guiza S (2017). Biosorption of heavy metal from aqueous solution using cellulosic waste orange peel. Ecol Eng.

[16] Hosseini SM, Sohrabnejad S, Nabiyouni G, Jashni E, Van der Bruggen B, Ahmadi A (2019). Magnetic cation exchange membrane incorporated with cobalt ferrite nanoparticles for chromium ions removal via electrodialysis. J Membr Sci.

[17] Hussin F, Aroua MK, Roziki MZ, Yusoff R (2020). Carbon dioxide adsorption using biomass-based activated carbon functionalized with deep eutectic solvents. IOP Conf Ser Mater Sci Eng.

[18] Kaur G, Singh N, Rajor A (2021). Efficient adsorption of doxycycline hydrochloride using deep eutectic solvent functionalized activated carbon derived from pumpkin seed shell. ChemistrySelect.

[19] Kebede A, Kedir K, Melak F, Asere TG (2022). Removal of Cr(VI) from aqueous solutions using biowastes: tella residue and pea (*Pisum sativum*) seed shell. Sci World J.

[20] Khalil U, Shakoor MB, Ali S, Rizwan M (2018). Tea waste as a potential biowaste for removal of hexavalent chromium from wastewater: equilibrium and kinetic studies. Arab J Geosci.

[21] Kotas J, Stasicka Z (2000). Chromium occurrence in the environment and methods of its speciation. Environ Pollut.

[22] Krishna RH, Swamy AV (2012). Studies on removal of Cr (VI) from aqueous solutions using powder of Mosambi fruit peelings (PMFP) as a low cost sorbent. E-J Chem.

[23] Kumar A, Thakur A, Panesar PS (2019). Extraction of hexavalent chromium by environmentally benign green emulsion liquid membrane using tridodecyamine as an extractant. J Ind Eng Chem.

[24] Leng L, Xu S, Liu R, Yu T, Zhuo X, Leng S, Xiong Q, Huang H (2019). Nitrogen containing functional groups of biochar: an overview. Bioresour Technol.

[25] Li C, Huang C, Zhao Y, Zheng C, Su H, Zhang L, Luo W, Zhao H, Wang S, Huang L (2021). Effect of choline-based deep eutectic solvent pretreatment on the structure of cellulose and lignin in bagasse. Processes.

[26] Li J, Ma J, Guo Q, Zhang S, Han H, Zhang S, Han R (2020). Adsorption of hexavalent chromium using modified walnut shell from solution. Water Sci Technol.

[27] Liang L, Wang Q, Xi F, Tan W, Zhang YT, Cheng LB, Wu Q, Xue YY, Meng X (2020). Effective removal of Cr(VI) from aqueous solution using modified orange peel powder: equilibrium and kinetic study. Nat Environ Pollut Technol.

[28] Lin C, Luo W, Luo T, Zhou Q, Li H, Jing L (2018). A study on adsorption of Cr (VI) by modified rice straw: characteristics, performances and mechanism. J Clean Prod.

[29] Lin D, Wu FC, Hu Y, Zhang T, Liu C, Hu Q, Hu Y, Xue Z, Han H, Ko T (2020). Adsorption of dye by waste black tea powder: parameters, kinetic, equilibrium, and thermodynamic studies. J Chem.

[30] López-Ramón MV, Stoeckli F, Moreno-Castilla C, Carrasco-Marín F (1999). On the characterization of acidic and basic surface sites on carbons by various techniques. Carbon.

[31] Ma Y, Liu W, Zhang N, Li Y, Jiang H, Sheng G (2014). Polyethylenimine modified biochar adsorbent for hexavalent chromium removal from the aqueous solution. Bioresour Technol.

[32] Nguyen TC, Nguyen XT, Tran DM, Vu QT, Nguyen VH, Nguyen DT, Do M, Nguyen TL, Ly T, Thai H (2020). Adsorption ability for toxic chromium (VI) ions in aqueous solution of some modified oyster shell types. Bioinorg Chem Appl.

[33] Ozcan AS, Ozcan AS, Tunali S, Akar T, Kıran İ (2005). Determination of the equilibrium, kinetic and thermodynamic parameters of adsorption of copper (II) ions onto seeds of Capsicum annuum. J Hazard Mater.

[34] Patiño J, Gutiérrez MC, Carriazo D, Ania CO, Fierro JL, Ferrer ML, Monte FD (2014). DES assisted synthesis of hierarchical nitrogen-doped carbon molecular sieves for selective CO_2_ versus N_2_ adsorption. J Mater Chem.

[35] Peng H, Guo J, Li B, Liu Z, Tao C (2018). High-efficient recovery of chromium (VI) with lead sulfate. J Taiwan Inst Chem Eng.

[36] Peng H, Guo J (2020). Removal of chromium from wastewater by membrane filtration, chemical precipitation, ion exchange, adsorption electrocoagulation, electrochemical reduction, electrodialysis, electrodeionization, photocatalysis and nanotechnology: a review. Environ Chem Lett.

[37] Sirviö JA, Visanko M, Liimatainen H (2015). Deep eutectic solvent system based on choline chloride-urea as a pre-treatment for nanofibrillation of wood cellulose. Green Chem.

[38] Smith EL, Abbott AP, Ryder KS (2014). Deep eutectic solvents (DESs) and their applications. Chem Rev.

[39] Tsamo C, Djomou Djonga PN, Dangwang Dikdim JM, Kamga R (2017). Kinetic and equilibrium studies of Cr(VI), Cu(II) and Pb(II) removal from aqueous solution using red mud, a low-cost adsorbent. Arab J Sci Eng.

[40] Xie Bingbing. Experimental study on treatment of Cr(VI) in wastewater by Tieguanyin Tea Stalks. Master dissertation, Chongqing University at Chongqing. 2018.

[41] Ye S, Xiong W, Liang J, Yang H, Wu H, Zhou C, Du L, Guo J, Wang W, Xiang L, Zeng G, Tan X (2021). Refined regulation and nitrogen doping of biochar derived from ramie fiber by deep eutectic solvents (DESs) for catalytic persulfate activation toward non-radical organics degradation and disinfection. J Colloid Interface Sci.

[42] You S, Yun H, Liu X, Wei C (2018). Synergetic removal of Pb(II) and dibutyl phthalate mixed pollutants on Bi_2_O_3_-TiO_2_ composite photocatalyst under visible light. Appl Catal B Environ.

[43] Yu W, Wang C, Yi Y, Zhou W, Wang H, Yang Y, Tan Z (2019). Choline chloride-based deep eutectic solvent systems as a pretreatment for nanofibrillation of ramie fibers. Cellulose.

[44] Zhang H, Lang J, Lan P, Yang H, Lu J, Wang Z (2020). Study on the dissolution mechanism of cellulose by ChCl-based deep eutectic solvents. Materials.

